# Characteristics of Effective Antitachycardia Pacing for Ventricular Tachycardia: The Importance of True Septal Lead Position

**DOI:** 10.1016/j.jacasi.2025.07.005

**Published:** 2025-08-22

**Authors:** Satoshi Yanagisawa, Yasuya Inden, Yuki Sato, Ryo Watanabe, Hiroyuki Miyazawa, Kiichi Miyamae, Tomoya Iwawaki, Takayuki Goto, Shun Kondo, Masaya Tachi, Masafumi Shimojo, Yukiomi Tsuji, Takahiro Okumura, Toyoaki Murohara

**Affiliations:** a Department of Advanced Cardiovascular Therapeutics, Nagoya University Graduate School of Medicine, Nagoya, Japan; b Department of Cardiology, Nagoya University Graduate School of Medicine, Nagoya, Japan; c Department of Clinical Engineering, Nagoya University Hospital, Nagoya, Japan

**Keywords:** antitachycardia pacing, cardiac resynchronization therapy defibrillator, implantable cardioverter-defibrillator, true septum pacing, ventricular tachycardia

## Abstract

**Background:**

Antitachycardia pacing (ATP) has a potential benefit for shock reduction of implantable cardioverter-defibrillator (ICD) recipients; however, its clinical utility and characteristics are unknown.

**Objectives:**

This study aims to extract characteristics leading to a high ventricular tachycardia (VT) termination rate of ATP.

**Methods:**

Patients who had a history of ≥1 ATP treatment episode from ICD or cardiac resynchronization therapy–defibrillator (CRTD) devices were included. All ATP treatments wherein intracardiac electrograms could be traced were reviewed. Two endpoints of VT termination were defined: type-I break (termination with 0-1 beat) and clinical endpoint of termination (≤5 beats). We assessed the characteristics associated with a high success rate of ATP using the logistic regression generalized estimating equation method.

**Results:**

Of 756 recipients using high-power devices, 1,468 treatment episodes in 119 patients were analyzed. The VT rate of <188 beats/min (vs ≥188 beats/min), CRTD (vs ICD), and true septum right ventricular lead position were significantly associated with high success rate of type-I break termination (generalized estimating equation success rate: 78.7% vs 64.7%, *P* = 0.011; 80.1% vs 66.5%, *P* = 0.021; and 79.8% vs 60.5%, *P* = 0.023, respectively). True septum lead position and slow VT were also independently associated with successful termination with clinical endpoint. The termination rate was highest in the right ventricular true septum position across all positions at both endpoints. The pacing QRS interval was significantly shorter in the septum group than in the nonseptum group (166.2 ± 21.9 ms vs 198.7 ± 26.5 ms; *P* < 0.001).

**Conclusions:**

True septum lead position, in addition to slow VT and CRTD, may be key to high ATP termination success.

An implantable cardioverter-defibrillator (ICD) is an established treatment for terminating lethal arrhythmias of ventricular tachycardia (VT) and ventricular fibrillation (VF), and improving the prognosis in patients with structural heart disease for primary and secondary prevention.^1^, ^2^, ^3^ An ICD treatment is comprised of shock therapy and antitachycardia pacing (ATP). The efficiency of ICD treatment leading to improvement in mortality has been mostly driven by shock therapy;^4^ however, ICD shock treatment itself was significantly associated with worsening prognosis. Multiple ICD shock burdens carry the risk of anxiety and depression post-treatment, which necessitates the need for shock reduction and improvement of patient quality of life.^4^^,^^5^

An ATP option is typically used in the ICD treatment program before shock delivery, and several ventricular pacing stimulus, such as burst or ramp pacing and newly developed pacing maneuvers, terminated VTs with considerable success rates.^6^, ^7^, ^8^, ^9^, ^10^, ^11^, ^12^ Previous retrospective studies have shown the possible benefit of ATP for improving mortality rates and heart failure hospitalization compared to shock therapy.^13^^,^^14^ In addition, ATP treatment did not affect daily physical activity or increased the risk of depression and anxiety, unlike shock therapy;^5^ however, there was a risk of VT acceleration after rapid ventricular pacing of ATP, causing faster VT development and incoming shock treatment. Thus, patients who can receive the maximum benefit of ATP should be identified in clinical settings, and these patients should be programmed with the most effective ATP sequence options. Currently, few studies have evaluated the characteristics associated with a high success rate of ATP,^10^^,^^15^ and no definitive parameters have been developed.

To explore the aforementioned hypothesis, the present study was conducted to extract clinical characteristics leading to the high VT termination rate of ATP in ICD recipients using a large-scale database of ICD treatments with more than 1,000 records, and to evaluate the difference in the success rate of ATP across detailed patient characteristics from various perspectives.

## Methods

### Study design and population

The study population comprised of patients who had a history of ≥1 ATP treatment episode from ICD or cardiac resynchronization therapy–defibrillator (CRTD) devices at the Nagoya University Hospital, Japan, between January 2017 and December 2023. All ATP treatments that were appropriately applied to treat VT or fast VTs in the VT zone and episodes where intracardiac electrograms could be fully traced during the treatment record were included. ATP treatments for VF episodes (before and during the charge period) were also analyzed. The interrogation records and electrograms at the time of ICD treatment were prospectively stored in the hospital data system, and all detailed episodes of the patients were retrospectively made available on demand, if applicable. Patients who received only shock treatment and those with no ATP treatment episodes or electrocardiograms stored in the interrogation system were excluded. The indications for ICD and CRTD implantation were in accordance with the guidelines.^16^ All patients received dual-chamber ICDs, and all screw leads were deployed using an inner stylet system. During the implantation procedure, right ventricular (RV) leads were aimed to be implanted in the septal position using fluoroscopy guidance as much as possible. The Institutional Ethics Committee at Nagoya University Hospital approved the study’s protocol (approved No. 2015-0192). Written informed consent was obtained from all the patients before the procedure. This study complied with the principles of the Declaration of Helsinki.

### Programming of ATP treatment and zone setting

The ICD treatment was uniformly programmed in most patients at our institution. Typically, the therapeutic zones for the treatment were set at 400 ms (150 beats/min), 320 ms (188 beats/min), and 270 ms (222 beats/min) for the VT, fast VT, and VF zones, respectively. In most cases, we set 2 zones, VT and VF, but in cases with a history of previously detected fast VT, an additional fast VT zone was programmed. The therapy zone was adjusted for some patients with specific VT rates by attending physicians and clinical engineers, if applicable. The number of intervals to detect the VT and VF was programmed to be 32 and 30/40 beats, respectively. For the VT zone, a maximum of 3 burst sequences were delivered, followed by 3 ramp pacing and 3 burst pacing when the VT persisted. Burst pacing consisted of 12 pacing pulses with an R-S1 reduction interval of 91% and a decrease in the pacing interval of 10 ms for every sequence. Ramp pacing consisted of 6 pulses with an R-S1 reduction interval of 94% and a decrement of 10 ms. If ATP failed to terminate VT, shock therapy was programmed using low- to high-voltage energy. For fast VTs, 2 sequences of burst pacing were programmed, followed by cardioversion. In rapid tachycardia assigned to the VF zone, 1- to 2-burst pacing was delivered during charging and before shock therapy. The novel intrinsic ATP algorithm was also programmed, if applicable, as previously reported by our laboratory team.^12^ The details of the ICD treatment programs are summarized in Supplemental Table 1. All ATP treatments were administered through the RV pacing lead. The minimum pacing interval of ATP was set to 160 and 200 ms for intrinsic ATP and conventional ATP, respectively. Supraventricular tachyarrhythmia discrimination, such as onset, stability, PR-logic, and wavelet analysis, was programmed to “on” in the VT or VF zone of the dual-chamber device. All inappropriate ATP treatments for supraventricular tachycardia were excluded based on a closed review of the electrograms. Acceleration was defined as a decrease of >10% in the cycle length (CL) of the VT or a change to another VT with a different waveform on the electrogram after ATP.

### Data collection and outcome measurements

Two endpoints of VT termination were defined: type-I break, which was successful VT termination with 0–1 beat after ATP, and clinical endpoint of termination, which was successful VT termination with ≤5 beats after ATP. We reviewed all the electrograms of the study population, and the mean VT rate, number of sequence deliveries, types of ATP, and minimum pacing interval in each episode were collected. Patient characteristics, medical history, and examination results were obtained from hospital medical records. At each VT episode on different days in the same patient, the patient age, medication information, therapeutic history, and examination results were individually updated as closely as possible before the corresponding episode. The history of catheter ablation, device information, administration of beta-blockers and class III antiarrhythmic drugs, and echocardiography data were reviewed as the most recent data before treatment for all patients. All patients receiving an ICD or CRTD were followed up at the outpatient clinic every 1 to 6 months after implantation. Device interrogation was performed every 6 to 8 months. A remote-monitoring system was used to detect ventricular arrhythmias in all patients. In case of an alarm due to the occurrence of VT or VF and their associated treatments in the monitoring system, we encouraged patients to visit the hospital immediately to assess their condition and provide them with a detailed therapeutic course using further examinations.

We compared the success rate of ATP treatment across different patient characteristics and VT episodes, and elucidated the independent factors associated with a high success rate of ATP for VT termination in univariable and multivariable analyses. To overcome the effects of repeated VT episodes in the same patient, success rates and outcomes were adjusted using the logistic regression generalized estimating equation (GEE) method. The rates of acceleration and shock therapy were assessed using the same maneuvers.

The cutoff level for fast VT was defined as 188 beats/min (320 ms), based on previous large-scale studies.^6^^,^^10^ The detailed position of the RV lead was assessed using imaging modalities, such as computed tomography and echocardiography with multiple cross-sectional images that were previously evaluated for any purpose in all patients. We classified the RV lead position into 5 categories: true septum, septal hinge, free wall, inferior hinge, and apex. The septal hinge was defined as the boundary between the septum and the free wall, and the inferior hinge was defined as the boundary between the septum and the inferior free wall. All lead positions were determined using computed tomography and echocardiography, with classification based on insertion site and distal tip of the lead. Most lead placements in 111 (93%) patients were assessed using computed tomography with thin-slice imaging, whereas the remaining patients were evaluated using echocardiography imaging. For echocardiographic assessment, 2 independent investigators who were blinded to patient outcomes and characteristics evaluated lead positions using multiplanar views. If there was disagreement, the final location was determined by consensus after thorough discussion.

Paced QRS duration was measured via surface electrocardiography under stable pacing conditions or during pacing stimulation testing. During ATP, paced QRS duration was measured using far-field intracardiac electrograms (Can to RV coil) stored in the episode records.^17^ For each patient, the first VT episode with available near-field (RV tip to ring) and far-field recordings was selected for comparison.^17^

### Statistical analysis

Continuous variables were expressed as mean ± SD or median (Q1-Q3), and categorical values were expressed as numbers (percentages). Comparisons of the differences in the baseline characteristics were analyzed using a Student’s *t*-test and the Mann–Whitney *U* test for parametric and nonparametric data, respectively. Pearson’s chi square and Fisher exact tests were used to compare categorical variables, as appropriate. The endpoints of ATP success rate were adjusted using the logistic regression GEE method. Using a generalized linear logistic model, the trends in the GEE-estimated success rates across continuous variables were calculated. For GEE logistic regression analysis, variables with *P* < 0.05 in the univariable analysis were entered in the GEE multivariable logistic regression model to identify independent predictors. This analysis evaluated the individual effects of each significant parameter on successful ATP outcomes, accounting for interactions among variables through multiple multivariable models. To assess the independent contribution of each factor, a multivariate analysis was performed using all pairwise combinations of the parameters. Because the outcome variable was binary, the GEE model followed a binomial distribution. A logit link function and exchangeable working correlation structure were used in the analysis. The data were assumed to be missing at random. Additionally, the model’s fit and performance were evaluated using the quasi-likelihood information criterion. Statistical significance was set at *P* < 0.05. All analyses were performed using SPSS version 28.0 (SPSS Inc) and R (version 4.3.3).

## Results

### Baseline characteristics

Among a total of 756 patients with ICD and CRTD who were followed up during the study period at our institution, 1,468 treatment episodes in 119 patients were analyzed. The baseline characteristics of the patients at the time of their first ATP episode are shown in Table 1. The mean age was 66.7 ± 14.3 years, and 78% were male. Fifty-seven (48%) patients underwent ICD implantation. The mean left ventricular (LV) ejection fraction was 37.8% ± 14.0%, and 32 (27%) patients had ischemic heart disease. The mean VT rate was 156.8 ± 30.7 beats/min with a mean of 1.47 ± 1.39 ATP sequences delivered. The therapeutic zone has been additionally adjusted in 15 (13%) patients to target a specific VT rate, primarily aiming for a lower rate below 150 beats/min. In addition, 12 patients (10%) had changes in ATP setting and sequence order following VT episodes that were not successfully terminated by initial ATP attempts during the study period.

**Table 1:** Baseline Characteristics and VT Episodes in the Eligible Study Population (1,468 Episodes in 119 Patients)

Patient characteristics^a^	
Age, y	66.7 ± 14.3
Male	93 (78)
Body mass index, m/kg^2^	23.3 ± 5.1
ICD/CRTD	57 (48)/62 (52)
Antiarrhythmic drugs class III^b^	67 (56)
Beta-blockers	107 (90)
Left ventricular ejection fraction, %	37.8 ± 14.0
Left ventricular endo-diastolic diameter, mm	59.5 ± 11.0
History of VT ablation	65 (55)
Number of VT ablations	1.0 ± 1.3
RV lead septum	82 (69)
Number of VT treatment episodes	4.0 (1.0–12.0)
Time from implant to first treatment, mo	42.7 (12.2–83.0)
Underlying cardiomyopathy and etiology	
Ischemic heart disease	32 (27)
Dilated cardiomyopathy	33 (28)
Hypertrophic cardiomyopathy	23 (19)
Sarcoidosis	11 (9.2)
Valvular disease	3 (2.5)
Amyloidosis	2 (1.7)
Others	15 (13)
VT characteristics	
Mean VT rate, beats/min	156.8 ± 30.7
Number of ATP sequences delivered, times	1.47 ± 1.39
Minimal pacing interval of ATP, ms	338.8 ± 70.1

Values are mean ± SD, median (Q1-Q3), or n (%).

ATP = antitachycardia pacing; CRTD = cardiac resynchronization therapy–defibrillator; ICD = implantable cardioverter-defibrillator; RV = right ventricular; VT = ventricular tachycardia.

a At the first instance of VT therapy during the study period.

b Class III antiarrhythmic drugs included amiodarone in 55 patients, sotalol in 11 patients, and a combination of amiodarone and sotalol in 1 patient.

### Success rate of ATP and predictors for VT termination

**Figure 1:**
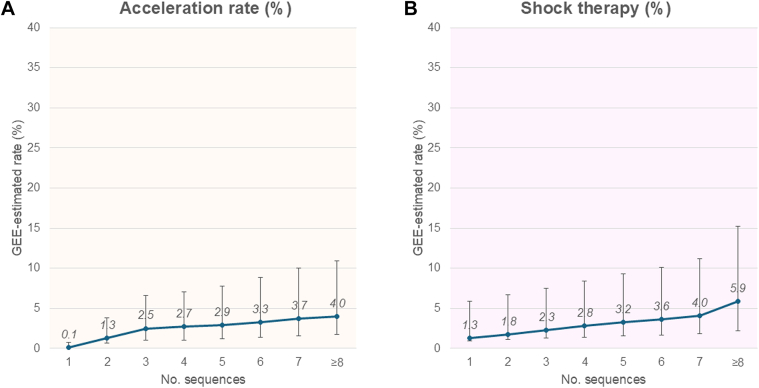
Cumulative Risk of Acceleration and Shock Across the ATP Sequences The generalized estimating equation (GEE)–estimated rates of acceleration (A) and shock therapy (B) according to the number of antitachycardia pacing (ATP) sequences. Error bars indicate 95% CIs.

The GEE-estimated ATP success rate for the type-I break and clinical endpoint of termination (≤5 beats) was 74.2% (95% CI: 68.5%-79.1%) and 84.5% (95% CI: 79.5%-88.5%), respectively. Acceleration rate and shock therapy in the GEE analyses were estimated as 4.0% (95% CI: 2.3%-6.9%) and 5.9% (95% CI: 3.7%-9.4%), respectively, and these risks incrementally increased along with the increased ATP sequences delivered (Figure 1).

**Figure 2:**
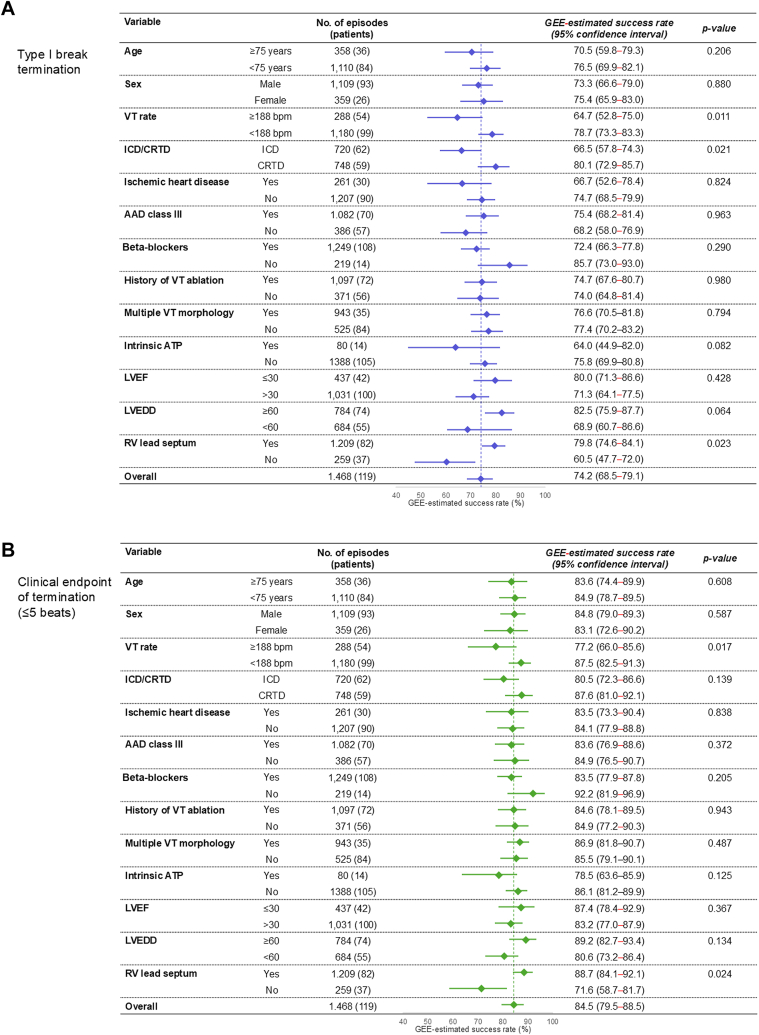
Characteristics Associated With Successful ATP for VT Termination (A) Type-I break. (B) Clinical endpoint (≤5 beats). Success rate was adjusted using the GEE method. At each ventricular tachycardia (VT) episode on different days in the same patient, the patient age, medication information, therapeutic history, and examination results were individually updated before the corresponding episode. The subgroup analysis comparing different ATP maneuvers (conventional ATP vs intrinsic ATP) was based on the initial programmed ATP setting at the beginning of the observational period. AAD = antiarrhythmic drug; CRTD = cardiac resynchronization therapy–defibrillation; ICD = implantable cardioverter-defibrillator; LVEDD = left ventricular end-diastolic diameter; LVEF = left ventricular ejection fraction; RV = right ventricle; VT = ventricular tachycardia; other abbreviations as in Figure 1.

For the endpoint of the type-I break, the univariable analysis of possible baseline characteristics and VT episodes associated with a high success rate of termination is shown in Figure 2A. The VT rate of <188 beats/min (vs ≥188 beats/min), CRTD (vs ICD), and true septum (vs nonseptum) RV lead position were significantly associated with high success rates of termination (GEE-estimated success rate: 78.7% [95% CI: 73.3%-83.3%] vs. 64.7% [95% CI: 52.8%-75.0%], *P* = 0.011; 80.1% [95% CI: 72.9%-85.7%] vs. 66.5% [95% CI: 57.8%-74.3%], *P* = 0.021; and 79.8% [95% CI: 74.6%-84.1%] vs 60.5% [95% CI: 47.7%-72.0%], *P* = 0.023, respectively) (Figure 2A).

In contrast, the univariable analysis of the high success rate of ATP for the clinical endpoint of termination (≤5 beats) demonstrated that VT rate of <188 beats/min (vs ≥188 beats/min; GEE-estimated success rate: 87.5% [95% CI: 82.5%-91.3%] vs 77.2% [95% CI: 66.0%-85.6%]; *P* = 0.017) and true septum (vs nonseptum) RV lead position (88.7% [95% CI: 84.1%-92.1%] vs 71.6% [95% CI: 58.7%-81.7%]; *P* = 0.024) were significantly associated with successful ATP outcome (Figure 2B).

The subsequent multivariable model for successful ATP treatment with type-I break including the 3 significant parameters demonstrated that all 3 factors, namely VT rate of ≥188 beats/min (odds OR: 0.544; 95% CI: 0.346-0.857; *P* = 0.009), CRTD (OR: 1.888; 95% CI: 1.104-3.210; *P* = 0.020), and true septum lead position (OR: 2.208; 95% CI: 1.163-4.192; *P* = 0.015) were independently associated with successful ATP outcome (Table 2). All predictors remained significant in the different multivariable models when one factor was excluded, indicating that these three factors independently affected ATP success (Table 2). The VT rate of ≥188 beats/min (OR: 0.465; 95% CI: 0.278-0.776; *P =* 0.003), and true septum lead position (OR: 1.832; 95% CI: 1.122-2.991; *P* = 0.015) were also independently associated with successful termination with clinical endpoint (Table 2). These independent parameters remained significant after adjusting for age and sex in the multivariate models (Table 2).

**Table 2:** GEE-Multivariable Models of Predictors for Successful ATP Treatment With Type-I Break and Clinical Endpoint^a^

Endpoints and Multivariable Model Pattern	Odds Ratio	95% CI	*P* Value	QIC
Type-I break (0–1 beat)				
Model 1				1,381.96
VT rate ≥188 beats/min	0.544	0.346-0.857	0.009	
CRTD (vs ICD)	1.888	1.104-3.210	0.020	
RV lead septum	2.208	1.163-4.192	0.015	
Model 2				1,396.53
VT rate ≥188 beats/min	0.559	0.366-0.855	0.007	
CRTD (vs ICD)	1.869	1.125-3.104	0.016	
Model 3				1,398.52
CRTD (vs ICD)	1.835	1.083-3.110	0.024	
RV lead septum	2.280	1.211-4.292	0.011	
Model 4				1,382.92
VT rate ≥188 beats/min	0.562	0.351-0.900	0.016	
RV lead septum	2.111	1.072-4.160	0.031	
Model 5				1,375.07
VT rate ≥188 beats/min	0.477	0.312-0.729	<0.001	
CRTD (vs ICD)	1.595	1.092-2.328	0.016	
RV lead septum	1.589	1.053-2.400	0.028	
Clinical endpoint of termination with ≤5 beats				
Model 6				1,003.67
VT rate ≥188 beats/min	0.462	0.275-0.777	0.004	
RV lead septum	1.724	1.056-2.815	0.029	
Model 7				1,004.96
VT rate ≥188 beats/min	0.465	0.278-0.776	0.003	
RV lead septum	1.832	1.122-2.991	0.015	

CRTD = cardiac resynchronization therapy defibrillator; ICD = implantable cardioverter-defibrillator; GEE = generalized estimating equation; QIC = quasi-likelihood information criterion; other abbreviations as in Table 1.

a This analysis examined the individual effects of 3 parameters: VT rate ≥188 beats/min, CRTD (vs ICD), and RV lead septum, on successful ATP outcomes, while accounting for potential interactions among these variables using multivariable models. Because the endpoint variable was binary, the GEE method was applied using a binomial distribution. To independently assess the contribution of each parameter, a multivariable analysis was conducted using all pairwise combinations of the three variables. Model 1 included all 3 parameters, whereas models 2-4 included each pairwise combination. Model 6 included all significant parameters for the clinical endpoint in univariate analysis, whereas models 5 and 7 included all significant parameters in univariate analysis after adjusting for age and sex. All models showed statistically significant effects of the included parameters, indicating that each independently influenced the endpoint.

### Detailed analyses of predictors for ATP success

**Figure 3:**
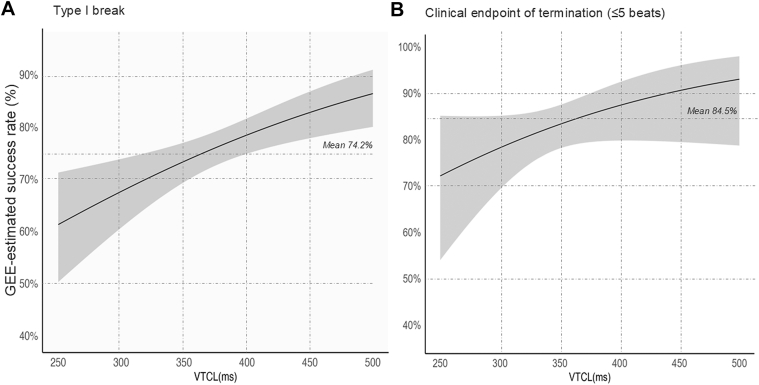
VT Termination Rate Across Tachycardia CL Termination with type-I break (A) and clinical endpoint (≤5 beats) (B). The success rate of VT termination was adjusted using the GEE method. CL = cycle length; VTCL = ventricular tachycardia cycle length; other abbreviations as in Figures 1 and 2.

Figure 3 shows the changes in the GEE-estimated ATP success rate of termination with type-I break and clinical endpoint across VT CL. The success rate incrementally increased with the prolonged VT CL in both endpoints of termination.

**Figure 4:**
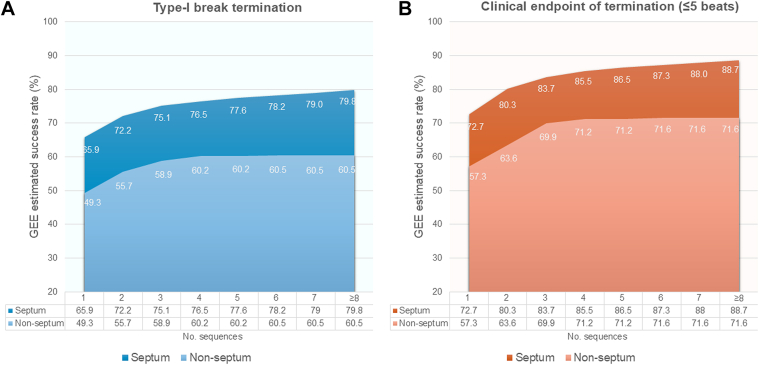
Cumulative Success Rate Between the Septal and Nonseptal Lead Positions Cumulative GEE-estimated success rate of termination with type-I break (A) and clinical endpoint of termination (≤5 beats) (B) across the number of ATP sequences. Abbreviations as in Figure 1.

**Figure 5:**
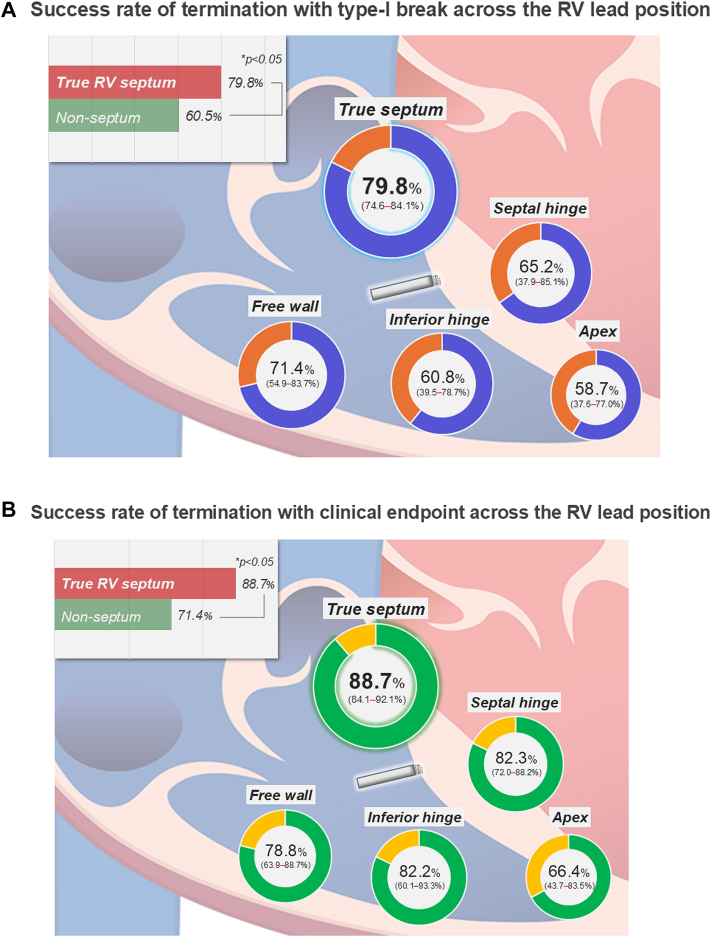
Success Rate of ATP Across the RV Lead Position Termination with type-I break (A) and clinical endpoint (≤5 beats) (B). The ATP success rates were adjusted using the GEE method. RV = right ventricular; other abbreviations as in Figure 1.

The cumulative success rate of ATP according to the number of sequences between the RV septum lead group and the nonseptum group revealed that the success rate was higher for every number of sequences in the septum group than in the nonseptum group, and these trends were observed even at the initial sequence of ATP (GEE-estimated success rate of termination with type-I break: 65.9% vs 49.3%, and clinical endpoint: 72.3% vs 57.3%, respectively) (Figure 4). The RV lead positions were the true septum in 82 (69%) patients, free wall in 16 (13%), apex in 11 (9.2%), inferior hinge in 7 (5.9%), and septal hinge in 3 (2.5%). Approximately 30% of the patients had RV lead implantation in the nonseptum position. The GEE-estimated success rate of termination with type-I break across the RV lead position was the highest in RV true septum (79.8%; 95% CI: 74.6%-84.1%), followed by free wall (71.4%; 95% CI: 54.9%-83.7%), septal hinge (65.2%; 95% CI: 37.9%-85.1%), inferior hinge (60.8%; 95% CI: 39.5%-78.7%), and apex (58.7%; 95% CI: 37.6%-77.0%), and the RV apex was the lowest success rate for VT termination (Figure 5A). Similarly, success rate of termination with clinical endpoint was the highest in RV true septum (88.7%; 95% CI: 84.1%-92.1%), followed by septal hinge (82.3%; 95% CI: 72.0%-88.2%), inferior hinge (82.2%; 95% CI: 60.1%-93.3%), free wall (78.8%; 95% CI: 63.9%-88.7%), and apex (66.4%; 95% CI: 43.7%-83.5%) (Figure 5B).

**Figure 6:**
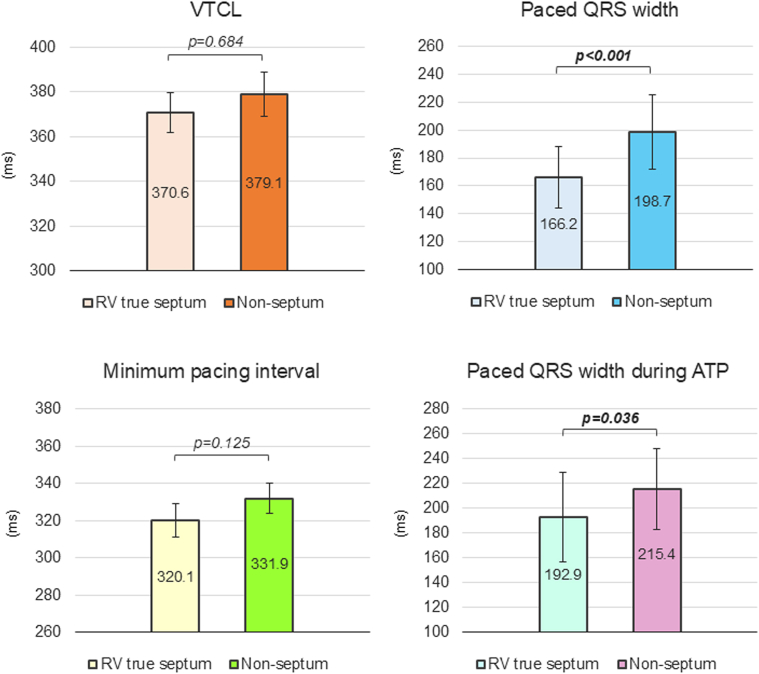
Clinical Parameters Between the Septal and Nonseptal Lead Position Groups The VTCL values and minimum pacing intervals were adjusted using the GEE method. The paced QRS width was compared between 42 patients in the RV septum group and 18 patients in the nonseptum group. The paced QRS duration during ATP was measured in the far-field intracardiac electrograms (Can to RV coil) stored in the episode record between the septal (n = 40) and nonseptal (n = 16) groups. The first VT episode in each patient, wherein both the near-field (RV tip to ring) and far-field (Can to RV coil) were available in the electrograms, was measured and compared.^17^ Examples were presented in Supplemental Figures 2 and 3. Abbreviations as in Figures 1, 3, and 5.

Comparison of the baseline characteristics between the RV lead septum and nonseptum groups demonstrated no significant differences in most characteristics except for the higher prevalence of history of catheter ablation for VT and number of the ablations in the septum group than in the non-septum group (50 [61%] patients vs 15 [41%], *P* = 0.038 and 1.2 ± 1.3 vs 0.7 ± 1.0, *P* = 0.041, respectively) (Supplemental Table 2). The VT CL (370.6 ± 9.0 ms vs 379.1 ± 9.8 ms, *P* = 0.684), number of the ATP sequences (1.46 ± 1.39 vs 1.56 ± 1.38, *P* = 0.295), and minimal pacing interval of ATP (320.1 ± 8.8 ms vs 331.9 ± 8.0 ms, *P* = 0.125) were not significantly different after the adjustment of GEE calculation between the septum and nonseptum groups. However, the paced QRS interval in the RV septum group (n = 42) was significantly shorter than that in the nonseptum group (n = 18) (166.2 ± 21.9 ms vs 198.7 ± 26.5 ms, *P* < 0.001) (Figure 6). Moreover, paced QRS interval during ATP measured on the stored far-field intracardiac electrograms of the ICD^17^ was significantly shorter in the RV septum group (n = 40) than nonseptum group (n = 16) (192.9 ± 36.1 ms vs 215.4 ± 32.8 ms, *P* = 0.036) (Figure 6). The distribution of ablation sites in patients undergoing catheter ablation for VT was balanced between the RV septal group (n = 50) and the nonseptal lead group (n = 15), as shown in Supplemental Figure 1. The prevalence of ablation sites in the LV and RV septum was observed in 25 patients (50%) in the RV septal group and 6 patients (40%) in the nonseptal lead group, with no significant difference (*P* = 0.496).

### Representative cases with RV lead implanted in the true septum and nonseptum, and ATP outcomes

A representative VT with a CL of 420 ms in a patient with an ICD and an RV lead in the true septum that was successfully terminated by the first-burst ATP sequence is shown in Supplemental Figure 2. Following the first burst pacing of 380 ms, the VT terminated with a type-I break, and far-field electrograms (Can to RV coil) demonstrated a pacing QRS width of 155 ms during ATP.^17^ The pacing QRS width on the 12-lead electrocardiography was measured as 184 ms.

In contrast, a VT with a CL of 360 ms, which was not successfully terminated by the first burst pacing, is shown in Supplemental Figure 3. A pacing interval of 320 ms in burst pacing did not terminate the VT, and the VT persisted with a slightly decreased CL after ATP. The pacing QRS width on the far-field electrograms was 229 ms, possibly suggesting a long electrical distance from the RV lead to the VT circuit. In this case, the RV lead was implanted in the RV apex according to the computed tomography imaging study, and the pacing QRS width on the 12-lead surface electrocardiography was wide at 224 ms.

### Success rate between CRTD vs ICD

The baseline characteristics of CRTD (n = 57) and ICD recipients (n = 62) are shown in Supplemental Table 3. Lower LV ejection fraction and larger LV endo-diastolic diameter were observed in the CRTD group than in the ICD group (31.0% ± 10.9% vs 43.9% ± 13.8%, *P* < 0.001 and 63.6 ± 11.3 mm vs 55.7 ± 9.2 mm, *P* < 0.001, respectively), despite higher success rate of ATP in the CRTD recipients. The prevalence of dilated cardiomyopathy and sarcoidosis was notable in the CRTD group, whereas ischemic heart disease and hypertrophic cardiomyopathy were common in the ICD group. The VT CL and minimal pacing intervals of ATP were not significant after adjusting for the GEE between the 2 groups.

A total of 60 inappropriate ATP episodes in 13 patients were excluded from the analysis. The estimated rate of inappropriate ATP, adjusted using the GEE method, was 1.7% (95% CI: 0.9%-2.9%) overall, and 1.8% (95% CI: 0.9%-3.4%) vs 2.7% (95% CI: 1.0%-7.6%) in the septal and nonseptal lead groups, respectively (*P* = 0.958).

## Discussion

This study aimed to extract the characteristics associated with a high success rate of ATP for VT termination via a detailed assessment of intracardiac electrograms in a large-scale sample of the device dataset. A slow VT rate of <188 beats/min, true septum RV lead position, and the CRTD device were independently associated with a high success rate of termination (Central Illustration). The slow VT and true septum lead position were associated with the increased success rates of termination with both type-I breaks and clinical endpoints. The true septal lead position had the highest success rate of ATP compared to other nonseptal positions, which accounted for approximately 70% of the patients. The paced QRS interval was significantly shorter in the RV septum group than in the nonseptum group.

**Central Illustration:**
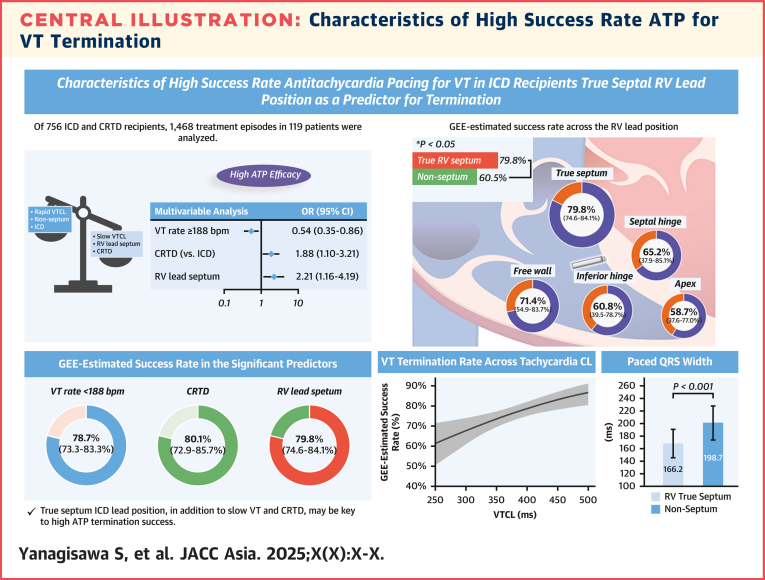
Characteristics of High Success Rate ATP for VT Termination A slow ventricular tachycardia (VT) rate of <188 bpm, true septum right ventricular (RV) lead position, and the cardiac resynchronization therapy–defibrillator (CRTD) device were independently associated with a high success rate of termination. The true septal lead position had the highest success rate of antitachycardia pacing (ATP) compared to other nonseptal positions, which accounted for approximately 70% of the patients. The paced QRS interval was significantly shorter in the RV septum group than in the nonseptum group. bpm = beats/min; CL = cycle length; GEE = generalized estimating equation; ICD = implantable cardioverter-defibrillator; VTCL = ventricular tachycardia cycle length.

The PainFREE Rx II (The Pacing Fast Ventricular Tachycardia Reduces Shock Therapies) study was the first clinical study to demonstrate the efficacy of ATP for terminating fast VT episodes with a 72% success rate using only 1 burst pacing.^6^ Since then, several studies have reported the success rate of ATP for fast VTs to be approximately 40% to 80%, and the results may have been influenced by the detection rate of VT, number of intervals to detect VTs, and programmed ATP characteristics.^6^, ^7^, ^8^, ^9^, ^10^ Moreover, ATP accompanied the risk of VT acceleration by up to approximately 10%,^6^^,^^8^, ^9^, ^10^ leading to unexpected shock therapy and decreased quality of life. Hence, increasing the number of ATP sequences to enhance the success rate of VT termination more frequently is not simple because it accompanies an increased risk of acceleration.^10^ Targeting specific characteristics associated with a high success rate of ATP is expected to focus on the intensive treatment of ATPs more effectively. Several factors which were responsible for the high termination rate after ATP have been reported in previous studies showing that beta-blocker administration and low heart rate preceding VT onset were independent predictors of ATP effectiveness.^15^^,^^18^ A recent subanalysis of the PainFree SmartShock Technology demonstrated that VT with a median CL of ≥320 ms had a significantly higher ATP success rate with 88.0% (95% CI: 84.8%-90.6%), which is consistent with our results. The slow rate of VT can be easily penetrated into the termination zone and refractory period of the VT circuit through a relatively long pacing interval without accelerating VT safely, and evidence regarding successful ATP for slow VT has proven this beneficial effect.^19^^,^^20^

Our study is unique because we found that the true septal lead position was an independent predictor of a high ATP success rate. Stimulation of the RV leads from the nonseptal position (ie, apex and free wall) provides abnormal electrical activation of the ventricular myocardium, which can increase the total ventricular activation time and delay contraction of the lateral wall of the LV. These conditions lead to further systolic dysfunction in the development of heart failure and increased mortality in patients with heart failure. An alternative lead position of the mid-interventricular septum has been proposed to obtain a narrower pacing QRS duration, possibly resulting in a reduction of LV activation dyssynchrony, heart failure hospitalization, and cardiac death.^21^, ^22^, ^23^ However, traditional approaches, such as stylet delivery and fluoroscopic guidance for lead deployment for the RV septum, are not always promising because a certain number of patients aimed at septal lead deployment were found to have a non-true septal lead position in a detailed assessment using post-imaging modality. A recent multicenter, randomized clinical study demonstrated that only 50% of the patients had successful RV lead deployment to the septum based on post-cardiac computed tomography using a classical, stylet delivery approach.^24^ These results were consistent to our findings that showed 31% of the patients had nonseptal lead deployment despite aiming for septal lead implantation during the procedure.

The advantage of true septal lead deployment is achieving narrow pacing QRS duration in comparison with nonseptal lead position, wherein the difference accounted for 13 ms on electrocardiography in a previous report.^22^ A decrease in the total ventricular activation time and shortened time to reach the lateral wall of the LV could be beneficial for ATP treatment to deliver stimulus activation earlier, which may result in minimizing the number of the S1 trains required to reach the VT circuit and entering the refractory period of VT.^25^ This speculation may be explained by the representative case of unsuccessful termination after ATP from the nonseptum lead position in Supplemental Figure 3, showing a possible long electrical distance from the pacing lead to the VT circuit, which might indicate that a greater number of S1 trains might be required to reach the VT circuit. Our findings contribute to the possible ATP treatment from a recently developed physiological pacing, namely the left bundle branch area pacing (LBBAP) lead implantation.^26^ Ponnusamy et al^27^ reported the feasibility of LBBAP lead implantation for sensing ventricular arrhythmias in 30 ICD recipients, with successful ATP terminating the VT of 75% (6 of 8 episodes) of patients and oversensing of the T-wave of 11%. The pacing QRS duration after successful LBBAP remarkably decreased from 164 to 126 ms compared to the typical apex pacing in another sensing study of LBBAP (CROSS-LEFT pilot study),^28^ which may suggest an easier ATP stimulus to reach the VT circuit with the possibility of high efficacy for termination. The LEADR trial demonstrated the feasibility and safety of a novel, lumenless, catheter-delivered, small-diameter (4.7-F) ICD lead, although all leads were implanted in the typical standard RV positions but not in the deep septal position, with the possibility of deep septal deployment for ATP as well as shock therapy in the future.^29^ In this context, a delivery catheter, which had the pre-shaped curve directed towards the septum orientation, used in LBBAP lead implantation had an advantage to have the RV lead guided towards the true septum more accurately.^24^ The fluoroscopic image using contrast injection through the side port of the catheter enhances the probability of the lead being placed in the true septum. Because septal lead deployment is the only manageable and controllable factor that could increase the success rate of ATP post-implantation, it is essential for operators to put every effort into implanting the RV lead in the true septum, which possibly leads to better patient outcomes. In addition to a delivery catheter with the ability to accommodate a newly developed small ICD lead (4.7-F),^29^ fluoroscopic projections with a steeper left anterior oblique angle exceeding 40 to 50 degrees and individualized left anterior oblique positioning technique can enhance the precision of true septum placement.^30^

The reason for the higher termination success rate in patients with CRTDs compared to those with ICD remains unclear, even though LV ejection fraction and end diastolic diameter were significantly worse in the CRT group. VT CL after GEE adjustment was not significantly different between the two groups. Differences in the prevalence of underlying cardiomyopathies, such as higher rates of dilated cardiomyopathy and sarcoidosis and lower rates of ischemic heart disease and hypertrophic cardiomyopathy in the CRTD group, may explain this finding, as previous studies have reported variations in ATP efficacy between nonischemic and ischemic cardiomyopathies.^6^^,^^31^^,^^32^ In contrast, comparative outcomes of ATP efficacy in patients with hypertrophic cardiomyopathy and cardiac sarcoidosis have been scarcely studied.^33^^,^^34^ Beyond differences in etiology, factors such as reverse remodeling and VT origin may also influence ATP success. However, the prevalence of septal ablation sites was similar between groups: 48% (n = 13 of 27) in the CRTD group and 47% (n = 13 of 38) in the ICD group (*P* = 0.951), indicating the need for further investigation to clarify the underlying mechanisms.

### Study limitations

This was a retrospective single-center study with a relatively small sample size of 119 patients, which may introduce selection bias and confounding in the analysis. Although we generalized the effect of repeated VT occurrences in the same patient using GEE analysis to calculate the success rate, we could not completely exclude the possibility dominated by patient characteristics because the number of VT incidences varied per patient. The number of S1 trains in the burst and ramp pacing of the ATP differed slightly from the typical pacing programs. Specifically, the 12 S1 pulses of the burst pacing adopted in the study were relatively greater than the typical eight trains; however, the reduction in the pacing interval was set to 91%, which was longer than the typical pacing interval of 88%, possibly indicating a similar extent of pacing intervention in the circuit. Computed tomography imaging was not originally obtained for the purpose of assessing lead position in this retrospective study, and in 8 patients (6.7%), lead position was assessed using echocardiography alone. This may have resulted in reduced positional accuracy.^35^ However, all electrocardiographic images were repetitively reviewed and confirmed by 2 independent investigators. We extracted as many characteristics associated with ATP success as possible, but there may be other unknown parameters that can increase the success rate in this population. The morphology of clinical VT and its site of origin may influence the success rate of ATP, alongside the pacing location. However, accurately matching VT characteristics induced during ablation with those recorded in device electrograms remains challenging. The paced QRS interval during ATP, as observed in stored far-field intracardiac electrograms, was obtainable in only approximately half of the study population because of the limited availability of both Can-to-coil and tip-to-ring configurations at the time of VT occurrence. ATP programs were individually modified in some patients based on VT rate and episode characteristics following termination failure. These adjustments, which were not accounted for in the statistical analysis, may have affected the reported success rate.

## Conclusions

True septal deployment of the RV lead may be promising for a high ATP success rate after ICD implantation in addition to a slow VT rate and having CRTD recipients. True septal lead implantation has safety benefits and potentiates the efficiency of ATP treatment, possibly minimizing shocks, and improvement of patients' quality of life.

## Funding Support and Author Disclosures

Drs Yanagisawa and Okumura are affiliated with a department sponsored by Medtronic Japan. All other authors have reported that they have no relationships relevant to the contents of this paper to disclose.
