# Supplementary material for: Characteristics of Effective Antitachycardia Pacing for Ventricular Tachycardia: The Importance of True Septal Lead Position
Source: JACC Asia. 2025 Aug 22;5(10):1329–43. doi: 10.1016/j.jacasi.2025.07.005 (PMC12790063; doi:10.1016/j.jacasi.2025.07.005)
Supplement: Supplemental Tables 1-3 and Supplemental Figures 1-3 [file mmc1.docx]

**Supplemental appendix**

Yanagisawa *et al.*: Characteristics of effective antitachycardia pacing for ventricular tachycardia: The importance of true septal lead position

**Table of contents**

**Page 2:** *Supplemental Table 1.* Detailed program settings for ICD implantation.

**Page 3-4:** *Supplemental Table 2.* Baseline characteristics and VT episodes in patients with and without true RV septum.

**Page 5-6:** *Supplemental Table 3.* Baseline characteristics and VT episodes in the CRT-D and ICD groups.

**Page 7:** *Supplemental Figure 1.* Distributions of the effective ablation site in patients undergoing catheter ablation for VT

**Page 8-9:** *Supplemental Figure 2.* A representative case where VT was successfully terminated by the first burst pacing in the RV lead septal group.

**Page 10-11:** *Supplemental Figure 3.* A representative case where VT was not terminated after ATP in the non-septal group.

**Supplemental Table 1**. Detailed program settings for ICD implantation.

| **Parameters** | **Typical therapeutic zone** | **Rx1** | **Rx2** | **Rx3** |
| --- | --- | --- | --- | --- |
| VF zone | 270 ms (222 bpm) | Burst (1–2) Shock^*^ | Shock | Shock |
| (Fast VT zone)** | 320 ms (188 bpm) | Burst (2) 12 pulses | CV | CV |
| VT zone | 400 ms (150 bpm) | Burst (3) 12 pulses^†^ | Ramp (3) 6 pulses^†^ | Burst (3) 12 pulses^†^ |

(Number of sequence) Number of pulses.

*ATP was delivered during or before shock therapy. **A fast VT zone was set as an option in patients with a history of previously detected rapid VT. ^†^The reductions in the pacing intervals were 91%, 94%, and 88% with a decrease in pacing interval of 10–20 ms for the first burst, ramp, and second burst pacing, respectively.

ATP, antitachycardia pacing; CV, cardioversion, ICD, implantable cardioverter defibrillator; VF, ventricular fibrillation; VT, ventricular tachycardia.

**Supplemental Table 2.** Baseline characteristics and VT episodes in patients with and without true RV septum.

| **Parameters** | **True RV septum**  **1,209 episodes in**  **82 patients** | **Non-septum**  **259 episodes in**  **37 patients** | **p-value** |
| --- | --- | --- | --- |
| **Patient characteristics*** |  |  |  |
| Age (years) | 67.7 ± 13.6 | 64.7 ± 15.7 | 0.285 |
| Male | 60 (73%) | 33 (89%) | 0.050 |
| Body mass index (m/kg^2^) | 23.3 ± 5.4 | 23.4 ± 4.3 | 0.941 |
| ICD/CRTD | 39 (48%)/43 (52%) | 18 (49%)/19 (51%) | 0.912 |
| Antiarrhythmic drugs class III | 46 (56%) | 21 (57%) | 0.946 |
| Β-blockers | 76 (93%) | 31 (84%) | 0.187 |
| Left ventricular ejection fraction | 37.0 ± 13.8 | 39.4 ± 14.6 | 0.392 |
| Left ventricular endo-diastolic diameter | 60.5 ± 11.4 | 57.2 ± 9.6 | 0.130 |
| History of VT ablation | 50 (61%) | 15 (41%) | 0.038 |
| Number of VT ablations | 1.2 ± 1.3 | 0.7 ± 1.0 | 0.041 |
| Number of VT treatment episodes | 4.0 (1.0–14.3) | 2.0 (1.0–6.5) | 0.074 |
| Time from implant to first treatment (months) | 42.5 (12.5–76.4) | 51.9 (10.8–89.9) | 0.951 |
| Underlying cardiomyopathy and etiology |  |  | 0.527 |
| Ischemic heart disease | 20 (24%) | 12 (32%) |  |
| Dilated cardiomyopathy | 26 (32%) | 7 (19%) |  |
| Hypertrophic cardiomyopathy | 14 (17%) | 9 (24%) |  |
| Sarcoidosis | 9 (11%) | 2 (5.4%) |  |
| Valvular disease | 2 (2.4%) | 1 (2.7%) |  |
| Amyloidosis | 2 (2.4%) | 0 (0%) |  |
| Others | 9 (11%) | 6 (16%) |  |
| **VT characteristics** |  |  |  |
| VT cycle length (ms) | 395.9 ± 70.5 | 399.4 ± 85.2 | 0.501 |
| Number of ATP sequences delivered | 1.46 ± 1.39 | 1.56 ± 1.38 | 0.295 |
| Minimal pacing interval of ATP (ms) | 336.1 ± 68.6 | 350.9 ± 75.5 | 0.002 |
| VT cycle length (ms)** | 370.6 ± 9.0 | 379.1 ± 9.8 | 0.684 |
| Minimal pacing interval of ATP (ms)** | 320.1 ± 8.8 | 331.9 ± 8.0 | 0.125 |

Data are presented as mean ± standard deviation, medians (interquartile ranges), or n (%).

*At the first instance of VT therapy during the study period. **Generalized estimating equation-estimated calculation.

ATP, antitachycardia pacing; CRTD, cardiac resynchronization therapy defibrillator; ICD, implantable cardioverter-defibrillator; VT, ventricular tachycardia.

**Supplemental Table 3.** Baseline characteristics and VT episodes in the CRT-D and ICD groups

| **Parameters** | **CRT-D group**  **748 episodes in**  **57 patients** | **ICD group**  **720 episodes in**  **62 patients** | **p-value** |
| --- | --- | --- | --- |
| **Patient characteristics*** |  |  |  |
| Age (years) | 68.6 ± 13.1 | 65.0 ± 15.3 | 0.170 |
| Male | 45 (79%) | 48 (77%) | 0.840 |
| Body mass index (m/kg^2^) | 22.4 ± 5.2 | 24.1 ± 4.9 | 0.069 |
| Antiarrhythmic drugs class III | 35 (61%) | 32 (52%) | 0.282 |
| Β-blockers | 49 (86%) | 58 (94%) | 0.170 |
| Left ventricular ejection fraction | 31.0 ± 10.9 | 43.9 ± 13.8 | <0.001 |
| Left ventricular end-diastolic diameter | 63.6 ± 11.3 | 55.7 ± 9.2 | <0.001 |
| History of VT ablation | 27 (47%) | 38 (61%) | 0.128 |
| Number of VT ablations | 0.9 ± 1.3 | 1.1 ± 1.2 | 0.430 |
| RV lead septum | 39 (68%) | 43 (69%) | 0.912 |
| Number of VT treatment episodes | 3.0 (1.0–16.0) | 4.0 (1.0–10.2) | 0.854 |
| Time from implant to first treatment (months) | 40.7 (12.6–80.6) | 49.2 (11.7–83.3) | 0.311 |
| Underlying cardiomyopathy and etiology |  |  | <0.001 |
| Ischemic heart disease | 12 (21%) | 20 (32%) |  |
| Dilated cardiomyopathy | 25 (44%) | 8 (13%) |  |
| Hypertrophic cardiomyopathy | 3 (5.3%) | 20 (32%) |  |
| Sarcoidosis | 9 (16%) | 2 (3.2%) |  |
| Valvular disease | 3 (5.3%) | 0 (0%) |  |
| Amyloidosis | 2 (3.5%) | 0 (0%) |  |
| Others | 3 (5.3%) | 12 (19%) |  |
| **VT characteristics** |  |  |  |
| VT cycle length (ms) | 387.5 ± 76.8 | 405.9 ± 68.1 | <0.001 |
| Number of ATP sequences delivered | 1.39 ± 1.30 | 1.56 ± 1.46 | 0.026 |
| Minimal pacing interval of ATP (ms) | 328.1 ± 73.1 | 349.9 ± 64.9 | <0.001 |
| VT cycle length (ms)** | 383.1 ± 8.3 | 369.2 ± 14.3 | 0.561 |
| Minimal pacing interval of ATP (ms)** | 328.7 ± 7.5 | 329.6 ± 13.2 | 0.393 |

*At the first instance of VT therapy during the study period. **Generalized estimating equation-estimated calculation.

ATP, antitachycardia pacing; CRTD, cardiac resynchronization therapy defibrillator; ICD, implantable cardioverter-defibrillator; VT, ventricular tachycardia.

**Supplemental Figure 1**

**
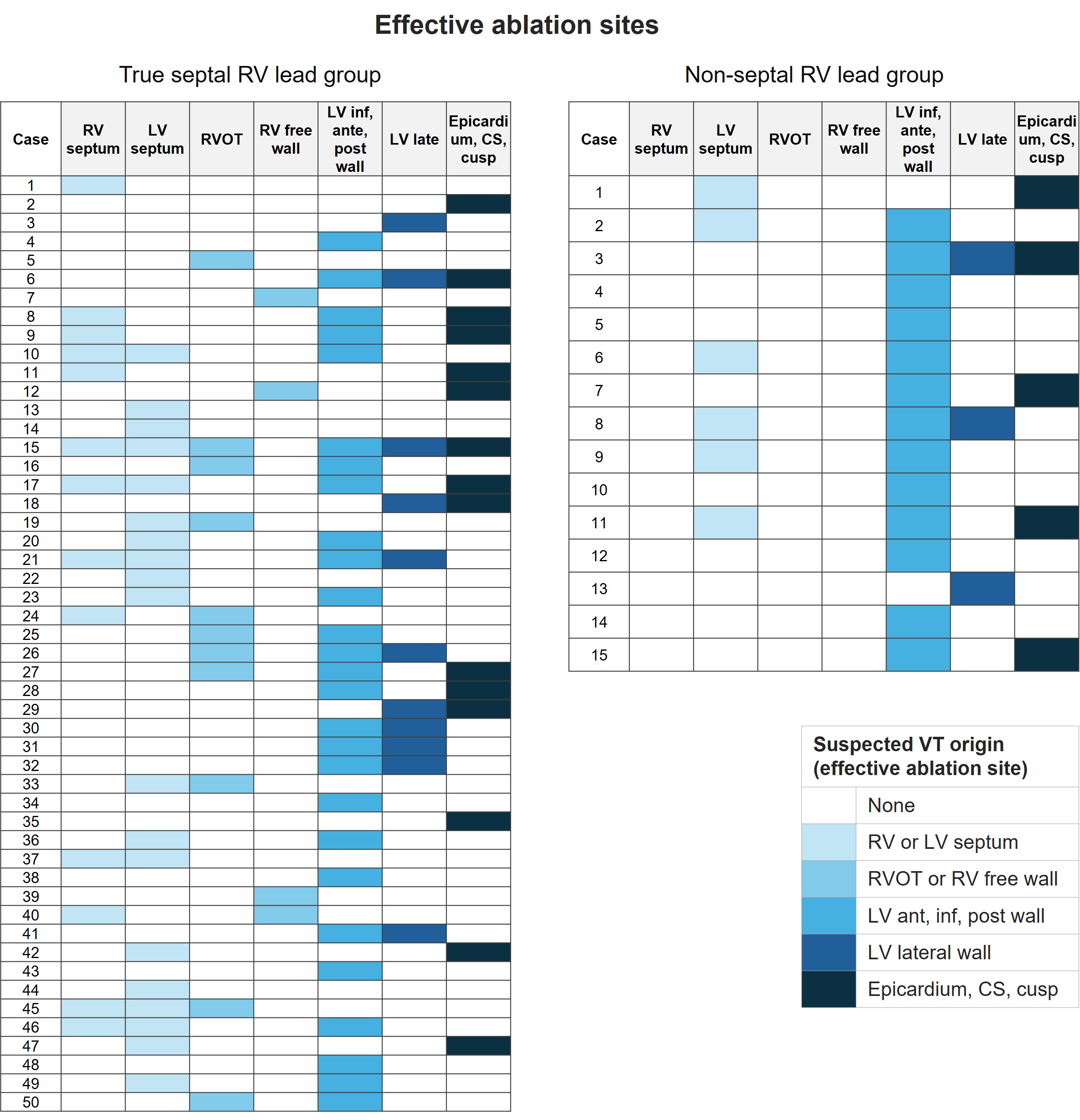
**

**Supplemental Figure 1. Distributions of the effective ablation site in patients undergoing catheter ablation for VT.**

Effective ablation sites were defined as the termination site of VT and estimated critical isthmus of VT circuit resulting in non-inducibility of target VTs following substrate ablation. Prevalence of the LV and RV septum ablation site was 25 (50%) patients and 6 (40%) in the RV septal and non-septal lead groups, respectively (*p*=0.496).

CS, coronary sinus; LV, left ventricular; RV, right ventricular; VT, ventricular tachycardia

**Supplemental Figure 2**
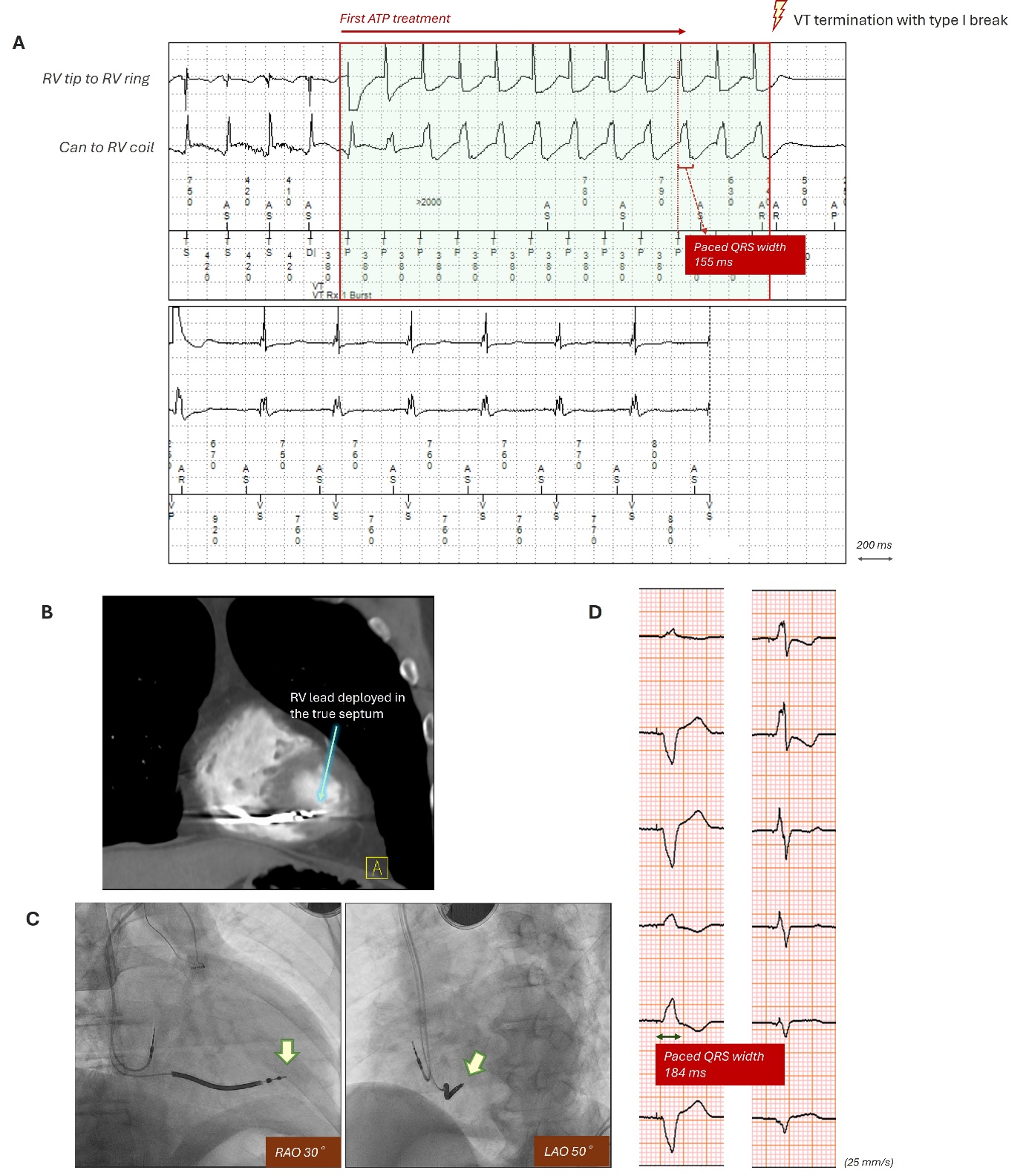


**Supplemental Figure 2. A representative case where VT was successfully terminated by the first burst pacing in the RV lead septal group.**

Intracardiac electrograms demonstrated that VT with a cycle length of 420 ms was successfully terminated by the first ATP with a pacing cycle length of 380 ms (A). The paced QRS duration on the far-field electrogram (Can to RV coil) during ATP was 155 ms. This case involved the implantation of an implantable cardioverter-defibrillator with the RV lead positioned at the true septum, which was confirmed using computed tomography (B) and fluoroscopy (C). The paced QRS duration in the RV lead captured using 12-lead electrocardiography was 184 ms (D). Notably, in the terminated VT example, the intrinsic QRS width appeared narrower than the paced QRS width during ATP. This observation suggests that the rapid global ventricular activation in clinical VT likely involved the septum or specialized conduction tissue, thereby enhancing VT termination during ATP delivered from the true septum.

ATP, antitachycardia pacing; LAO, left anterior oblique; RAO, right anterior oblique; RV, right ventricular; VT, ventricular tachycardia.

**Supplemental Figure 3**

**
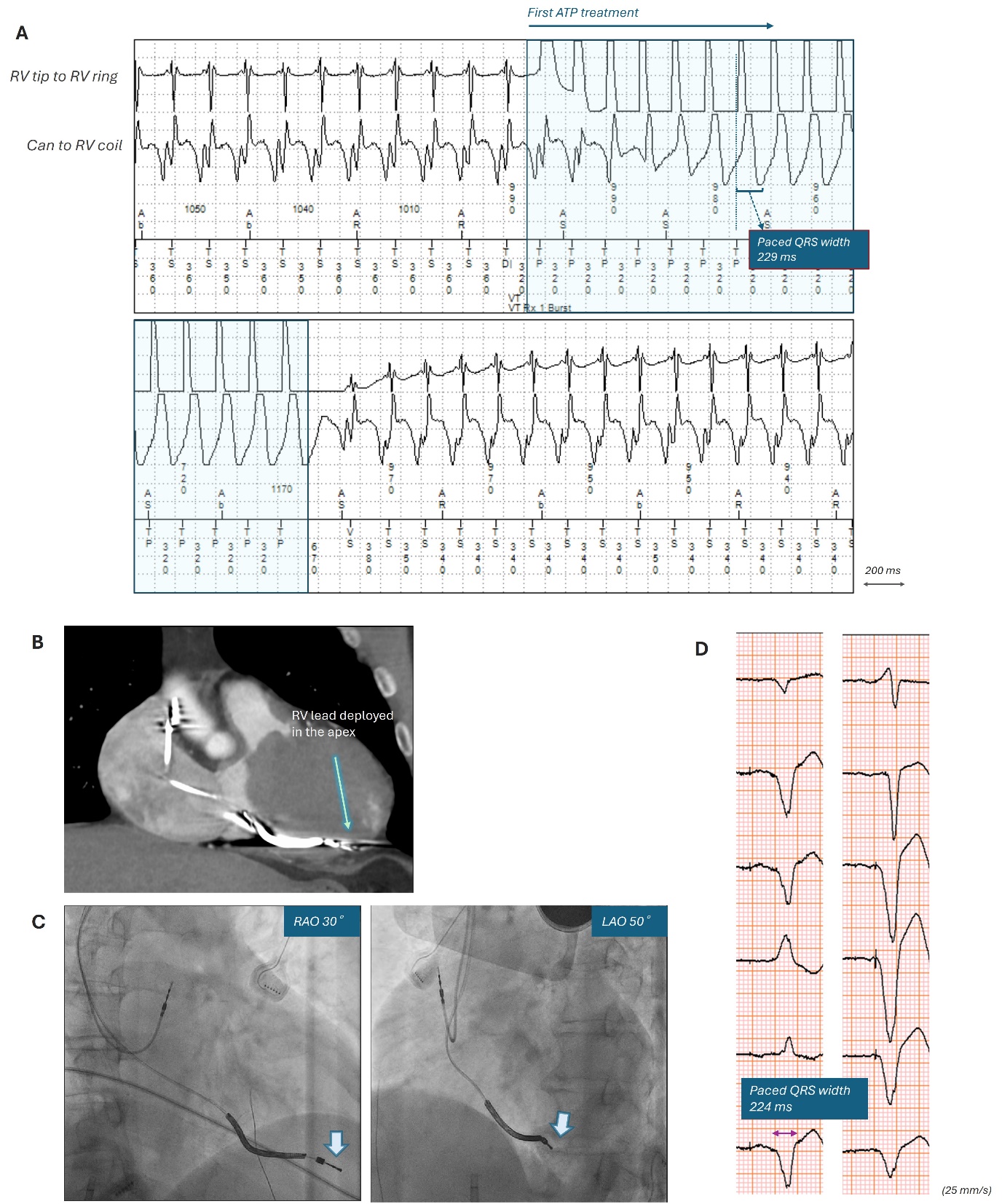
**

**Supplemental Figure 3. A representative case where VT was not terminated after ATP in the non-septal group.**

The VT CL was 360 ms. The pacing QRS width on the far-field electrograms was 229 ms, possibly suggesting a long electrical distance from the RV lead to the VT circuit. (A). The VT CL shortened to 340 ms after ATP. The RV lead with an implantable cardioverter-defibrillator was deployed to the apex, which was confirmed using computed tomography (B) and fluoroscopy (C). The 12-lead electrocardiography showed a paced QRS duration of 224 ms. (D). The wide intrinsic QRS width of the clinical VT on the electrograms was consistent with a distant site that did not engage the septum and might explain the failure to terminate.

ATP, antitachycardia pacing; CL, cycle length; RV, right ventricular; LAO, left anterior oblique; RAO, right anterior oblique; VT, ventricular tachycardia.
